# Autologous chondrocyte implantation, matrix‐induced autologous chondrocyte implantation, osteochondral autograft transplantation and osteochondral allograft improve knee function and pain with considerations for patient and cartilage defects characteristics: A systematic review and meta‐analysis

**DOI:** 10.1002/ksa.12525

**Published:** 2024-11-04

**Authors:** Joseph E. Nassar, Grace Guerin, Taidhgin Keel, Raffaella Russo, Filippo Familiari, Luke V. Tollefson, Robert F. LaPrade

**Affiliations:** ^1^ Department of Orthopedics Brown University Providence Rhode Island USA; ^2^ Faculty of Medicine American University of Beirut Medical Center Beirut Lebanon; ^3^ Department of Orthopedic Surgery Twin Cities Orthopedics Edina Minnesota USA; ^4^ Medical School University of Minnesota Minneapolis Minnesota USA; ^5^ Department of Medical and Surgical Sciences, Nutrition Unit Magna Graecia University Catanzaro Italy; ^6^ Department of Orthopaedic and Trauma Surgery Magna Graecia University Catanzaro Italy; ^7^ Research Center on Musculoskeletal Health Magna Graecia University Catanzaro Italy

**Keywords:** autologous chondrocyte implantation, matrix‐induced autologous chondrocyte implantation, osteochondral allograft, osteochondral autograft transplantation, patient reported outcomes

## Abstract

**Purpose:**

Previous studies have reported on the outcomes of autologous chondrocyte implantation (ACI) versus matrix‐induced ACI (MACI) and microfracture. Specific clinical outcomes of ACI, MACI, osteochondral autograft transplantation (OAT) and osteochondral allograft (OCA) have not been well studied. The purpose of this systematic review and meta‐analysis was to analyze the outcomes of these regenerative surgical techniques with an emphasis on comparing their effectiveness using the International Knee Documentation Committee (IKDC) subjective score, the Lysholm Knee Scoring Scale, the Tegner Activity Scale and the Visual Analogue Scale (VAS) score for the surgical treatment of tibiofemoral joint cartilage defects.

**Methods:**

An electronic search of MEDLINE, Embase and Cochrane Library was performed to identify studies that reported clinical outcomes for ACI, MACI, OAT and OCA procedures. The literature review was conducted following Preferred Reporting Items for Systematic Reviews and Meta‐Analyses guidelines and only studies involving cartilage defects in the tibiofemoral joint were included. Outcomes were measured with the IKDC evaluation, Lysholm Knee Scoring Scale, Tegner Activity Scale and the VAS. Outcomes were compared to the minimal clinically important difference (MCID) and patient acceptable symptom state (PASS). The methodological quality of the included studies was analyzed by the Methodological Index for Nonrandomized Studies and the Jadad scale.

**Results:**

Forty‐seven studies were included representing a total of 1993 patients with a mean follow‐up time of 57.2 ± 40.3 months (range: 4.0–160.0 months). The location of cartilage defects was reported in 46 studies, with a total of 1922 cartilage defects. There were 1277 medial femoral condyle cartilage defects, 488 lateral femoral condyle cartilage defects, 139 unspecified femoral condyle cartilage defects and 18 tibial plateau cartilage defects. All four procedures reported significant improvements in the Lysholm, IKDC, Tegner and VAS scores with no significant differences between them. The OAT technique surpassed the PASS threshold for the IKDC score while all four techniques surpassed the PASS threshold for Tegner and Lysholm scores. Additionally, all procedures met the MCID for each clinical outcome.

**Conclusion:**

This systematic review and meta‐analysis indicate that ACI, MACI, OAT and OCA all result in significant improvements in knee function and pain for cartilage defects of the tibiofemoral joint. When selecting a procedure, patient and cartilage defect characteristics should be assessed to determine the best technique for each individual patient.

**Study Design:**

Systematic review and meta‐analysis.

**Level of Evidence:**

Level III.

**Abbreviations**
ACI: autologous chondrocyte implantation
BMI: body mass index
IKDC: International Knee Documentation Committee
LFC: lateral femoral condyle
MACI: matrix‐induced autologous chondrocyte implantation
MCID: minimal clinically important difference
MFC: medial femoral condyle
MINORS: Methodological Index for Nonrandomized Studies
OAT: osteochondral autograft transplantation
OCA: osteochondral allograft
OCD: osteochondritis dissecans
PASS: patient acceptable symptom state
RoB: risk of bias
VAS: Visual Analogue Scale

## INTRODUCTION

The tibiofemoral joint is lined with articular cartilage which plays a crucial role in ensuring smooth joint movement [52]. Damage to this cartilage can occur for a variety of reasons, including trauma, abnormal joint loading or osteochondritis dissecans (OCD). This poses major clinical challenges due to the limited capacity of hyaline cartilage for self‐regeneration. If left untreated, cartilage defects can lead to progressive joint degeneration, chronic pain and functional impairment, often necessitating surgical intervention [5, 12, 41, 59]. Over time, many techniques have been developed and refined to address these defects, each reporting significant improvements in pain relief, joint stability and functional recovery. However, no consensus has been reached on the most optimal surgical procedure for treating cartilage defects of the tibiofemoral joint.

Regenerative techniques that promote hyaline cartilage formation include autologous chondrocyte implantation (ACI) and matrix‐induced ACI (MACI). Meanwhile, osteochondral autograft transplantation (OAT) and osteochondral allograft (OCA) involve transplant of an osteochondral unit. First introduced in the 1990s, ACI has demonstrated considerable success in restoring cartilage function but has several limitations such as the need for two operations and challenges achieving a watertight seal with periosteum or scaffolding material [9]. To address these drawbacks, MACI, also referred to as third‐generation ACI, was developed to be less invasive than previous generations with improved procedural efficiency. The OAT technique, commonly known as mosaicplasty, involves transplanting autologous osteochondral plugs from lesser‐weight‐bearing areas of the knee joint to the defect site. This allows for the simultaneous restoration of cartilage and subchondral bone, offering immediate structural stability [21]. However, its use is limited by the availability of donor grafts and the potential for donor site morbidity [32]. The OCA procedure has been developed to restore cartilage and underlying bone through the use of fresh or refrigerated allograft tissue. By using allograft cartilage, surgeons can access larger grafts without the risk of donor site morbidity [27].

Despite significant advancements in ACI, MACI, OAT and OCA, their comparative efficacy in treating tibiofemoral joint cartilage defects remains unclear. The purpose of this systematic review and meta‐analysis was to analyze the clinical outcomes of these regenerative surgical techniques with a focus on comparing their effectiveness using the International Knee Documentation Committee (IKDC) subjective score, the Lysholm Knee Scoring Scale, the Tegner Activity Scale and the Visual Analogue Scale (VAS) score for the surgical treatment of tibiofemoral joint cartilage defects. We hypothesize that all four procedures will result in improved clinical outcomes but the optimal technique may vary depending on the size and characteristics of the cartilage defects.

## METHODS AND MATERIALS

### Search strategy

This systematic review and meta‐analysis were performed in accordance with the Preferred Reporting Items for Systematic reviews and Meta‐Analyses guidelines. The study protocol was developed and registered at the International Prospective Register of Systematic Reviews (*CRD42024575008*).

A search was conducted over Medline, Embase and Cochrane Library from inception through 9 July 2024. The keywords ‘Autologous Chondrocyte Implantation’, ‘ACI’, ‘Matrix Associated Autologous Chondrocyte Implantation’, ‘Matrix Induced Autologous Chondrocyte Implantation’, ‘MACI’, ‘Osteochondral Autologous Transplantation’, ‘Osteochondral Autograft Transplantation’, ‘OAT’, ‘Osteochondral Allograft Transplantation’, ‘OCA’, ‘Tibiofemoral’, ‘Femoral Condyle’, ‘Tibial Plateau’ were used and combined using Boolean operators ‘AND’ and ‘OR’ to identify articles reporting on patient outcomes after undergoing ACI, MACI, OAT or OCA at the level of the tibiofemoral joint (Table 1). Additional relevant articles were then identified after reviewing the reference lists from included studies.

**Table 1:** Search strategy developed for this systematic review and meta‐analysis.

Search concept	Search terms
Procedures	(‘Autologous Chondrocyte Implantation*’ OR ‘ACI’
	(‘Matrix Associated Autologous Chondrocyte Implantation*’ OR ‘Matrix Induced Autologous Chondrocyte Implantation*’ OR ‘MACI’)
	(‘Osteochondral Autologous Transplantation*’ OR ‘Osteochondral Autograft Transplantation*’ OR ‘OAT’)
	(‘Osteochondral Allograft Transplantation*’ OR ‘OCA’)
Anatomical location	(‘Tibio?femoral*’ OR ‘Femoral Condyle*’ OR ‘Tibial Plateau*’)
Combined search strategy	(‘Autologous Chondrocyte Implantation*’ OR ‘ACI’ OR ‘Matrix Associated Autologous Chondrocyte Implantation*’ OR ‘Matrix Induced Autologous Chondrocyte Implantation*’ OR ‘MACI’ OR ‘Osteochondral Autologous Transplantation*’ OR ‘Osteochondral Autograft Transplantation*’ OR ‘OAT’ OR ‘Osteochondral Allograft Transplantation*’ OR ‘OCA’) AND (‘Tibio?femoral*’ OR ‘Femoral Condyle*’ OR ‘Tibial Plateau*’)

### Eligibility criteria

Inclusion criteria consisted of English and non‐English studies with level of evidence 1–4 that reported patient outcomes after either ACI, MACI, OAT or OCA for the treatment of articular cartilage defects of the tibiofemoral joint. Exclusion criteria consisted of review articles, editorial commentaries, case reports, biomechanical studies, epidemiological and database studies, studies reporting on patients with cartilage defects at the level of the patellofemoral joint, studies that included same patient data sets (the publication that had the most recent mean follow‐up was included) and studies with missing data. Three authors (JEN, GG, TK) independently and in duplicate conducted an initial title and abstract screening followed by a full‐text screening to determine articles that satisfied eligibility criteria and data extraction. A fourth independent author (LVT) was consulted to discuss and resolve any disagreements when present.

### Data extraction

Data were extracted from the included studies and tabulated using Microsoft Excel spreadsheet (Version 2007; Microsoft). Study characteristics from each article were extracted, including level of evidence, surgery type, sample size, patient demographics (age, sex, body mass index [BMI]), cartilage defects characteristics (number by type, size, cause) and patient follow‐up time. Preoperative and final follow‐up outcome measures including subjective Lysholm scores, IKDC scores, Tegner scores and general VAS scores were also extracted.

### Data and statistical analysis

Weighted means of patient‐reported outcomes at final follow‐up were calculated for the overall population of all included patients, as well as for subgroups based on the type of surgery (ACI vs. MACI vs. OAT vs. OCA). For the statistical analysis, Review Manager 5.4 (The Cochrane Collaboration) was used to calculate mean improvements from preoperative to postoperative levels of the extracted patient‐reported outcomes, reported as mean differences and the findings were presented using forest plots. Subgroup analyses were performed to compare outcomes between ACI, MACI, OAT and OCA. A 95% confidence interval (CI) and a random‐effects model were employed when considerable heterogeneity was observed, defined as *p* ≤ 0.05 on the *Q* test or *I*² > 50%. Weighted means were used for the continuous variables. A one‐way analysis of variance test was used to determine if there was any significant difference between treatment approaches, with statistical significance set to a *p* < 0.05. The minimal clinically important difference (MCID) is defined as the smallest change in a score that patients perceive as meaningful and sufficient to change their management. The patient acceptable symptom state (PASS) reflects the absolute postoperative score indicating satisfactory symptom levels used to assess clinical outcomes. These thresholds were extracted from the literature [1, 2, 3, 4].

### Risk of bias (RoB) assessment

Three authors (JEN, GG, TK) performed independently and in duplicate the quality assessment on all included studies. The Methodological Index for Nonrandomized Studies (MINORS) was used to assess the quality of nonrandomized studies. Any disagreements were resolved by a third author (initials blinded for review). The mean MINORS score was categorized based on the classification system by Ekhtiari et al. as very low for 0 < MINORS score < 6; low for 6 ≤ MINORS score < 10; fair for 10 ≤ MINORS score < 14 and good for MINORS score ≥ 14 [19]. The JADAD scale was also used to assess the quality of the included randomized studies categorized as low for JADAD scores ≤ 2 and high for JADAD scores ≥ 3.

## RESULTS

**Figure 1:**
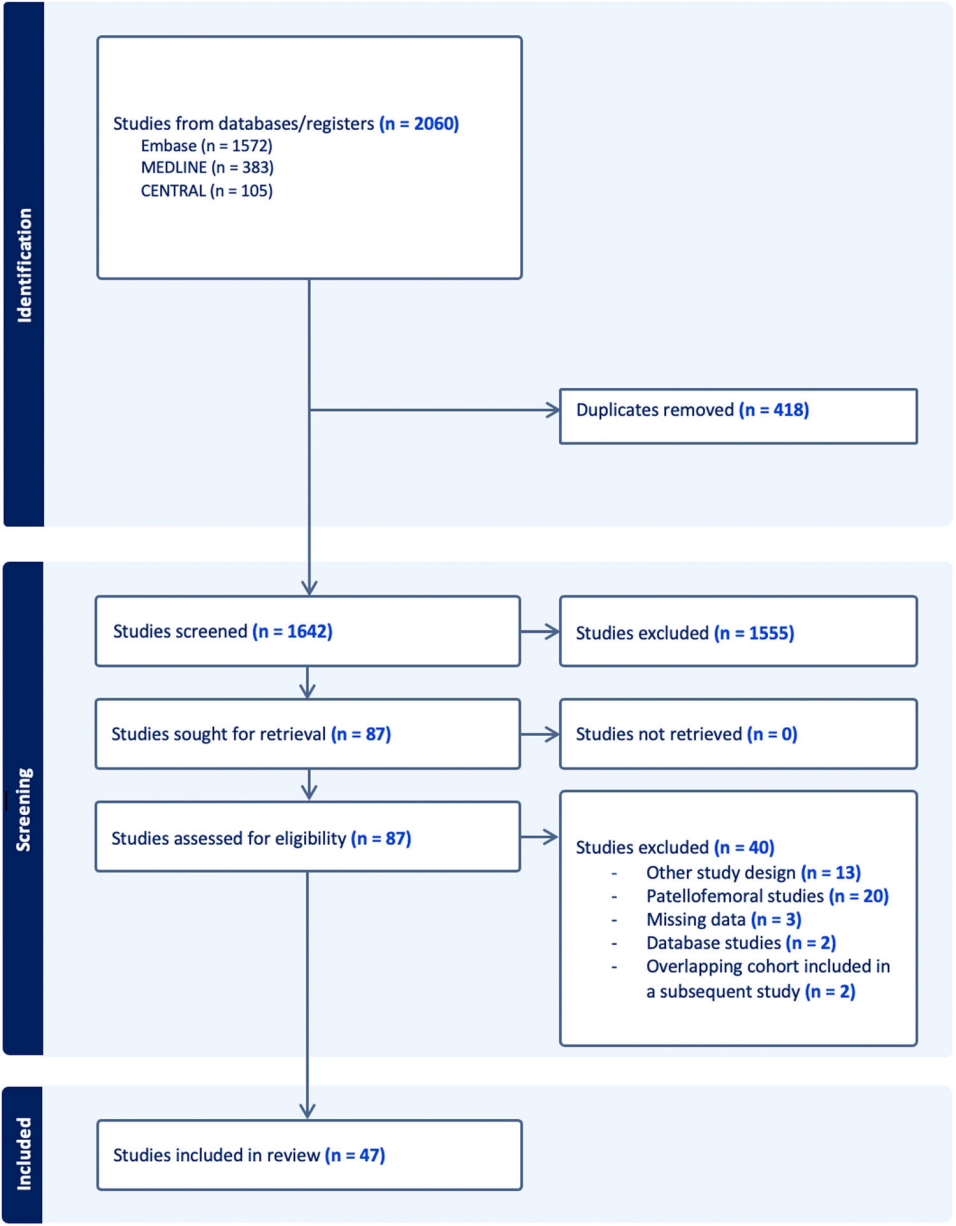
Preferred reporting items for systematic reviews and meta‐analyses flow diagram of the included studies.

The initial search identified 2060 articles. After removing duplicates, 1642 articles remained and were screened by title and abstract. A total of 88 articles progressed to the full‐text screening stage. Ultimately, 47 studies met the eligibility criteria and were included in the systematic review and meta‐analysis (Figure 1) [1, 3, 6, 7, 8, 10, 11, 13, 14, 15, 16, 17, 18, 20, 21, 22, 23, 24, 25, 26, 27, 29, 30, 31, 33, 35, 36, 37, 39, 40, 42, 44, 45, 46, 47, 48, 49, 50, 51, 52, 53, 54, 55, 56, 57, 58, 60, 61]. The mean level of evidence for these studies was 3.25 (range: 1–4) (Table 2).

**Table 2:** Summary of the included studies including level of evidence, surgery type, sample size, patients' demographics and follow‐up duration.

Author year	Level of evidence	Surgery type	Sample size	Mean age ± SD, years	Sex	Mean BMI ± SD, kg/m^2^	Mean follow‐up ± SD, months
**Abrams 2014** [1]	4	OCA	32	35.0 ± 10.0	17 M, 15 F	NR	52.8 ± 27.0
**Akgun 2015** [3]	2	MACI	14	32.5 ± 8.9	8 M, 6 F	NR	24.0
**Barie 2020** [6]	2	ACI	7	28.2 ± 9.1	7 M	25.4 ± 2.6	103.0 ± 9.6
		MACI	9	30.4 ± 6.8	6 M, 3 F	23.3 ± 1.2	115.0 ± 10.8
**Basad 2014** [7]	3	MACI	36	32.0 ± 9.0	NR	24.0 ± 3.0	24.0
**Bhattacharjee 2016** [8]	4	ACI	17	27.0 ± 7.0	NR	NR	60.0
**Brown 2011** [10]	2	OCA	45	34.5 ± 10.0	25 M, 9 F	26.9 ± 5.6	24.0
**Brown 2014** [11]	2	OCA	9	43.2 ± 15.6	6 M, 3 F	25.1 ± 4.0	24.0
**Chow 2004** [13]	4	OAT	30	44.6 ± 13.6	13 M, 17 F	NR	45.1 ± 11.3
**Clave 2016** [14]	1	MACI	30	29.2 ± 11.9	20 M, 10 F	NR	24.0
		OAT	30	29.2 ± 11.9	20 M, 10 F		19.0
**Crawford 2009** [15]	4	MACI	8	38.0 ± 6.1	5 M, 3 F	27.0 ± 5.2	26.0 ± 2.0
**Ebert 2012** [17]	4	MACI	20	34.0 ± 11.8	10 M, 10 F	26.3 ± 4.3	24.0
**Ebert 2017** [16]	1	MACI	17	36.4 ± 8.5	9 M, 9 F	26.2 ± 3.4	24.0
			18	36.4 ± 7.5	12 M, 7 F	25.2 ± 3.5	
**Ebert 2017** [18]	4	MACI	31	35.3 ± 11.8	15 M, 16 F	26.2 ± 4.7	60.0
**Emre 2013** [20]	4	OAT	152	24.8 ± 4.6	126 M, 26 F	NR	18.2 ± 4.2
**Erdil 2013** [21]	4	OAT	64	31.6 ± 11.6	47 M, 17 F	NR	82.2 ± 27.1
**Filardo 2011** [22]	4	ACI	62	28.1 ± 11.4	48 M, 14 F	NR	84.0
**Filardo 2014** [23]	3	OAT	31	32.5 ± 10.5	18 M, 13 F	24.0 ± 3.0	24.0
**Filardo 2015** [24]	4	OAT	26	29.3 ± 8.4	19 M, 7 F	23.0 ± 3.0	144.0
**Flohe 2011** [25]	3	MACI	20	35.0 ± 8.1	14 M, 6 F	NR	12.0
**Frank 2018** [26]	3	OCA	50	32.4 ± 10.3	27 M, 23 F	26.1 ± 4.4	5.1 ± 2.5
**Gilat 2021** [27]	4	OCA	160	31.9 ± 10.7	84 M, 76 F	26.7 ± 4.6	92.4 ± 32.4
**Giorgini 2013** [29]	4	OCA	11	34.1 ± 14.1	8, 3 F	NR	26.5
**Gudas 2012** [30]	1	OAT	57	24.3 ± 6.3	36 M, 21 F	NR	124.8 ± 6.5
**Gursoy 2021** [31]	3	OCA	14	30.1 ± 8.5	9 M, 5 F	26.7 ± 3.7	30.2 ± 4.7
			7	32.6 ± 10.2	6 M, 1 F	27.8 ± 3.7	30.3 ± 5.0
			6	27.2 ± 6.4	5 M, 1 F	24.8 ± 3.8	29.7 ± 4.9
**Hohmann 2016** [33]	4	OCA	9	32.1 ± 6.6	5 M, 4 F	NR	24.0
**Karataglis 2005** [35]	4	OCA	4	30.5 ± 8.6	2 M, 2 F	NR	33.0 ± 3.5
**Kon 2011** [36]	4	ACI	61	45.5 ± 4.9	40 M, 21 F	25.3 ± 2.7	60.0
**Kreuz 2013** [37]		ACI	28	35.6 ± 9.6	17 M, 11 F	25.0 ± 0.58	48.0
**LaPrade 2009** [39]	4	OCA	23	30.9 ± 8.8	13 M, 10 F	27.1 ± 4.4	36.0
**Lim 2012** [40]	2	ACI	18	25.1 ± 4.0	10 M, 8 F	NR	62.4 ± 14.5
		OAT	22	30.4 ± 5.5	12 M, 10 F		69.6 ± 14.9
**Ma 2004** [42]	4	OAT	18	29.0 ± 10.1	12 M, 6 F	NR	42.0 ± 11.5
**Marcacci 2007** [44]	4	OAT	20	29.3 ± 10.1	22 M, 8 F	NR	84.0
**McCulloch 2007** [45]	4	OCA	11	35.0 ± 9.2	18 M, 17 F	NR	35.0
**Ogura 2018** [47]	4	ACI	56	37.0 ± 11.0	34 M, 22 F	26.3 ± 4.3	99.6 ± 61.2
**Owusu 2021** [48]	3	OCA	91	42.6 ± 13.0	57, 34 F	25.9 ± 3.9	26.1
**Ozturk 2006** [49]	4	OAT	19	33.1 ± 6.8	13 M, 6 F	NR	32.4
**Peterson 2010** [50]	4	ACI	52	35.5 ± 13.0	NR	NR	153.6 ± 39.5
**Sasaki 2012** [51]	4	OAT	12	13.7 ± 1.3	10 M, 2 F	NR	26.2 ± 15.1
**Schneider 2011** [52]	4	MACI	81	32.5 ± 8.9	NR	24.5 ± 2.6	30.2 ± 17.4
**Scorrano 2004** [53]	3	ACI	4	24.8 ± 9.0	4 M	NR	24.0
**Stone 2014** [54]	3	OAT	7	23.7 ± 9.4	6 M, 1 F	NR	7.0 ± 3.6
**Takazawa 2012** [55]	2	ACI	10	33.7 ± 10.9	6 M, 4 F	NR	6.2 ± 0.3
**Tirico 2017** [56]	4	OCA	7	50.7 ± 5.0	5 M, 2 F	26.1 ± 2.2	97.1 ± 45.7
**Tirico 2018** [57]	3	OCA	156	29.6 ± 11.4	90 M, 53 F	25.0 ± 4.6	72.0 ± 39.6
**Tirico 2019** [58]	4	OCA	184	31.1 ± 11.7	175 M, 25 F	25.5 ± 4.9	80.4
**Wang 2017** [60]	3	OCA	50	35.0 ± 13.9	49 M, 28 F	NR	48.0
			27	35.0 ± 11.3			48.0
**Zak 2014** [61]	4	OAT	10	38.0 ± 8.4	8 M, 2 F	NR	84.0

Abbreviations: ACI, autologous chondrocyte implantation; BMI, body mass index; F, female; M, male; MACI, matrix‐associated autologous chondrocyte implantation; NR, not reported; OAT, osteochondral autologous transplantation; OCA, osteochondral allograft transplantation; SD, standard deviation.

### Patient characteristics

A total of 1993 patients were included in this study (Table 2). The mean patient age was 32.3 ± 11.4 years (range: 24.8–50.7 years). Mean ages were 34.4 ± 11.8 years for patients undergoing ACI, 33.2 ± 9.7 years for MACI, 28.7 ± 10.1 years for OAT and 33.2 ± 12.0 years for OCA (*p* < 0.05). Patient sex was reported in 43 studies (91.5%) (*n* = 1831/1993 patients) with 64.3% (*n* = 1194/1857) of those reported being male and 35.7% (*n* = 663/1857) being female. Patient BMI was reported in 22 studies (46.8%), with a mean BMI of 25.6 ± 4.3 kg/m² and a BMI range: 23–27.8 kg/m². Mean BMI was 25.6 ± 3.2 for ACI, 25.0 ± 3.5 for MACI, 23.5 ± 3.0 for OAT and 29.5 ± 4.6 for OCA (*p* < 0.05). The location of cartilage defects was reported in 46 studies (97.9%), with a total of 1922 cartilage defects (Table 3). Most cartilage defects occurred on the medial femoral condyle (1277 cartilage defects), followed by the lateral femoral condyle (488 cartilage defects), unspecified femoral condyle (139 cartilage defects) and the tibial plateau (18 cartilage defects). Cartilage defect sizes were reported in 38 studies (83.0%), with a mean cartilage defect size of 4.4 ± 3.6 cm² for patients undergoing ACI, 2.4 ± 3.9 cm² for patients undergoing MACI, 2.9 ± 1.4 cm² for patients undergoing OAT and 6.4 ± 4.3 cm² for patients undergoing OCA (*p* < 0.05). The aetiology of cartilage defects was reported in 31 studies (66.0%) (*n* = 1146/1922), with trauma being the leading cause (410 cartilage defects, 35.8%), followed by degenerative disease (367 cartilage defects, 32.0%), OCD (368 cartilage defects, 32.1%) and osteonecrosis (two cartilage defects, 0.2%). The mean follow‐up time was reported in all 47 studies (100.0%) and was 57.2 ± 40.3 months (range: 4.0–160.0 months). The mean follow‐up time was 85.1 ± 47.9 months for ACI, 31.8 ± 21.3 months for MACI, 54.9 ± 43.5 months for OAT and 59.7 ± 35.6 months for OCA (*p* < 0.05).

**Table 3:** Summary of cartilage defects' type, size, cause, previous surgery on the affected knee and concomitant procedures reported across the included studies.

Author year	Number of cartilage defects by type	Mean cartilage defects Size ± SD, cm^2^	Cause of cartilage defects	Previous surgery on affected knee	Concomitant procedures
**Abrams 2014** [1]	25 MFC, 8 LFC	4.7 ± 2.0	NR	NR	NR
**Akgun 2015** [3]	10 MFC, 4 LFC	3.0 ± 5.2	14 T	NR	0
**Barie 2020** [6]	7 MFC	4.1 ± 0.4	3 OCD, 4 D	7	NR
	8 MFC, 1 LFC	4.3 ± 0.2	5 OCD, 4 D	9	
**Basad 2014** [7]	24 MFC, 12 LFC	NR	65 T	NR	NR
**Bhattacharjee 2016** [8]	15 MFC, 2 LFC	4.5 ± 1.9	5 T, 12 OCD	17	NR
**Brown 2011** [10]	34 FC	5.7 ± 3.9	20 OCD, 15 D, 2 N	25	9
**Brown 2014** [11]	4 MFC, 5 LFC	3.4 ± 1.2	3 T, 3 OCD, 3 D	5	NR
**Chow 2004** [13]	28 MFC, 2 LFC	NR	17 T, 4 OCD, 9 D	10	13
**Clave 2016** [14]	30 FC	3.1 ± 0.8	NR	NR	NR
	25 FC	3.5 ± 0.3			
**Crawford 2009** [15]	6 MFC, 2 LFC	2.9 ± 1.4	8 T	6	NR
**Ebert 2012** [17]	11 MFC, 3 LFC, 6 TP	2.7 ± 5.2	NR	14	NR
**Ebert 2017** [16]	13 MFC, 5 LFC	3.2 ± 1.5	NR	NR	0
	14 MFC, 5 LFC	2.9 ± 1.9			0
**Ebert 2017** [18]	18 MFC, 7 LFC, 6 TP	2.5 ± 1.2	NR	18	NR
**Emre 2013** [20]	127 MFC, 25 LFC	2.7 ± 0.7	NR	NR	33
**Erdil 2013** [21]	40 MFC	NR	47 T, 18 D	0	NR
**Filardo 2011** [22]	45 MFC, 17 LFC	2.5 ± 1.8	29 T, 10 OCD, 23 D	35	35
**Filardo 2014** [23]	25 MFC, 6 LFC	NR	4 T, 11 OCD, 16 D	21	15
**Filardo 2015** [24]	17 MFC, 10 LFC	1.9 ± 0.6	6 T, 20 D	12	16
**Flohe 2011** [25]	16 MFC, 4 LFC	7.1 ± 1.8	12 T, 8 OCD	NR	NR
**Frank 2018** [26]	50 FC	NR	NR	29	NR
**Gilat 2021** [27]	90 MFC, 76 LFC	NR	78 T, 33 OCD, 49 D	155	95
**Giorgini 2013** [29]	2 MFC, 5 LFC, 4 TP	10.3 ± 5.8	4 T, 5 OCD, 2 D	NR	NR
**Gudas 2012** [30]	48 MFC, 11 LFC	NR	32 T, 25 OCD	NR	0
**Gursoy 2021** [31]	9 MFC, 5 LFC	NR	NR	NR	0
	5 MFC, 2 LFC				0
	3 MFC, 3 LFC				0
**Hohmann 2016** [33]	7 MFC, 2 LFC	8.1 ± 1.4	5 T, 4 OCD	2	NR
**Karataglis 2005** [35]	3 MFC, 1 LFC	9.1 ± 2.5	4 OCD	NR	0
**Kon 2011** [36]	54 MFC, 7 LFC	2.9 ± 1.2	6 T, 55 D	26	26
**Kreuz 2013** [37] **LaPrade 2009** [39]	16 MFC, 3 LFC	5.3 ± 2.6	NR	NR	NR
	12 MFC, 1 LFC	4.2 ± 1.3			
**Lim 2012** [40]	20 MFC, 4 LFC	4.8 ± 1.9	14 OCD, 9 D	20	NR
**Lim 2012** **Ma 2004** [42]	13 MFC, 5 LFC	2.8 ± 0.7	NR	NR	0
	19 MFC, 3 LFC	2.8 ± 0.9			0
**Marcacci 2007** [44]	11 MFC, 5 LFC, 2 TP	2.8 ± 1.1	18 T	6	10
**McCulloch 2007** [45]	17 MFC, 13 LFC	1.9 ± 0.2	NR	13	19
**Ogura 2018** [47]	22 MFC, 4 LFC	5.3 ± 2.3	26 OCD	13	NR
**Owusu 2021** [48]	32 MFC, 26 LFC	11.8 ± 8.0	NR	52	31
**Ozturk 2006** [49]	NR	NR	NR	60	10
**Peterson 2010** [50]	15 MFC, 4 LFC	7.1 ± 6.8	9 T, 2 OCD, 8 D	NR	15
**Sasaki 2012** [51]	41 MFC, 11 LFC	4.9 ± 3.9	NR	NR	54
**Schneider 2011** [52]	8 MFC, 4 LFC	2.7 ± 5.5	12 OCD	NR	NR
**Scorrano 2004** [53]	67 MFC, 14 LFC	5.4 ± 2.4	32 OCD, 84 D	95	29
**Stone 2014** [54]	2 MFC, 2 LFC	7.0 ± 3.6	4 T	3	NR
**Takazawa 2012** [55]	5 MFC, 2 LFC	3.3 ± 1.5	7 OCD	7	NR
**Tirico 2017** [56]	7 MFC, 3 LFC	3.5 ± 3.0	8 T, 2 D	4	2
**Tirico 2018** [57]	7 MFC	4.6 ± 0.9	7 N	6	NR
**Tirico 2019** [58]	101 MFC, 55 LFC	6.4 ± 2.1	NR	130	NR
**Wang 2017** [60]	138 MFC, 62 LFC	6.3 ± 2.2	28 T, 126 OCD, 46 D	172	NR
**Wang 2017** [61]	43 MFC, 34 LFC	5.8 ± 2.4	NR	26	13
**Zak 2014** [61]	7 MFC, 3 LFC	2.8 ± 1.5	8 T, 2 OCD	8	NR

Abbreviations: D, degenerative; LFC, lateral femoral condyle; MFC, medial femoral condyle; N, necrosis; NR, not reported; OCD, osteochondritis dissecans; SD, standard deviation; T, traumatic; TP, tibial plateau.

### Lysholm score

**Figure 2:**
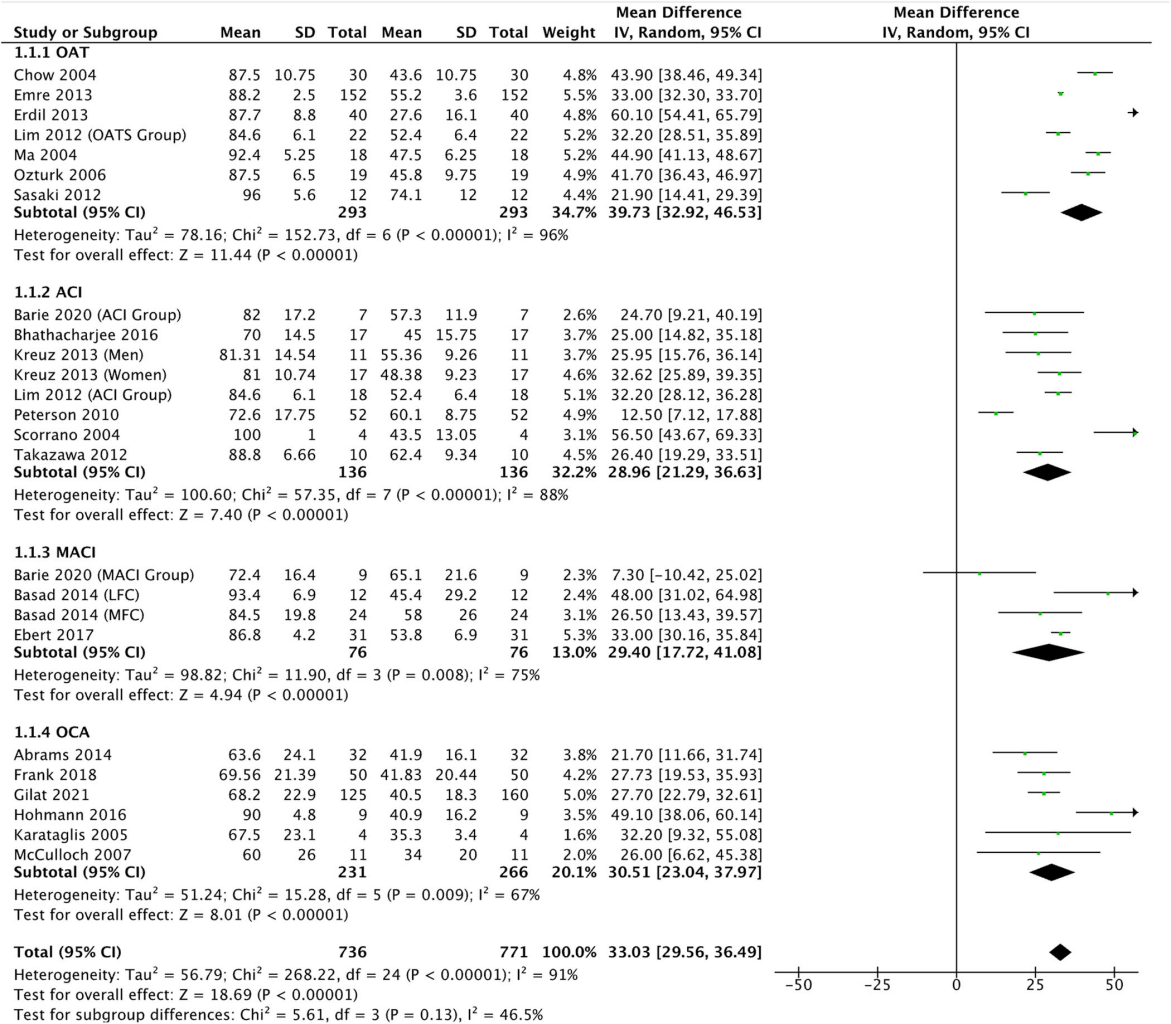
Forest plot showing mean improvement in Lysholm scores from preoperative to postoperative in patients treated for cartilage defects at the level of the tibiofemoral joint.

The postoperative Lysholm score was reported in 21 studies (44.7%), representing 736 patients, with a mean score at final follow‐up of 80.2 ± 18.3 (range: 60.0–96.0). Patients treated using ACI reported a mean score of 78.1 ± 15.8 (range: 70–88.8), those treated using MACI reported a mean score of 86.2 ± 13.0 (range: 72.4–93.4), those treated using OAT reported a mean score of 88.3 ± 6.1 (range: 84.8–96.0) and those treated using OCA reported a mean score of 68.3 ± 24.5 (range: 60.0–90.0). Patients undergoing ACI showed a 29.0 mean improvement [95% CI, 21.3, 36.6] (*p* < 0.05) from preoperative to postoperative Lysholm scores, those undergoing MACI showed a 29.4 point improvement [95% CI, 17.7, 41.1] (*p* < 0.05), those undergoing OAT showed a 39.7 point improvement [95% CI, 32.9, 46.5] (*p* < 0.05), while those undergoing OCA reported a 30.5 point mean improvement [95% CI, 23.0, 38.0] (*p* < 0.05) (*p*‐subgroup difference > 0.05) (Figure 2).

### IKDC score

**Figure 3:**
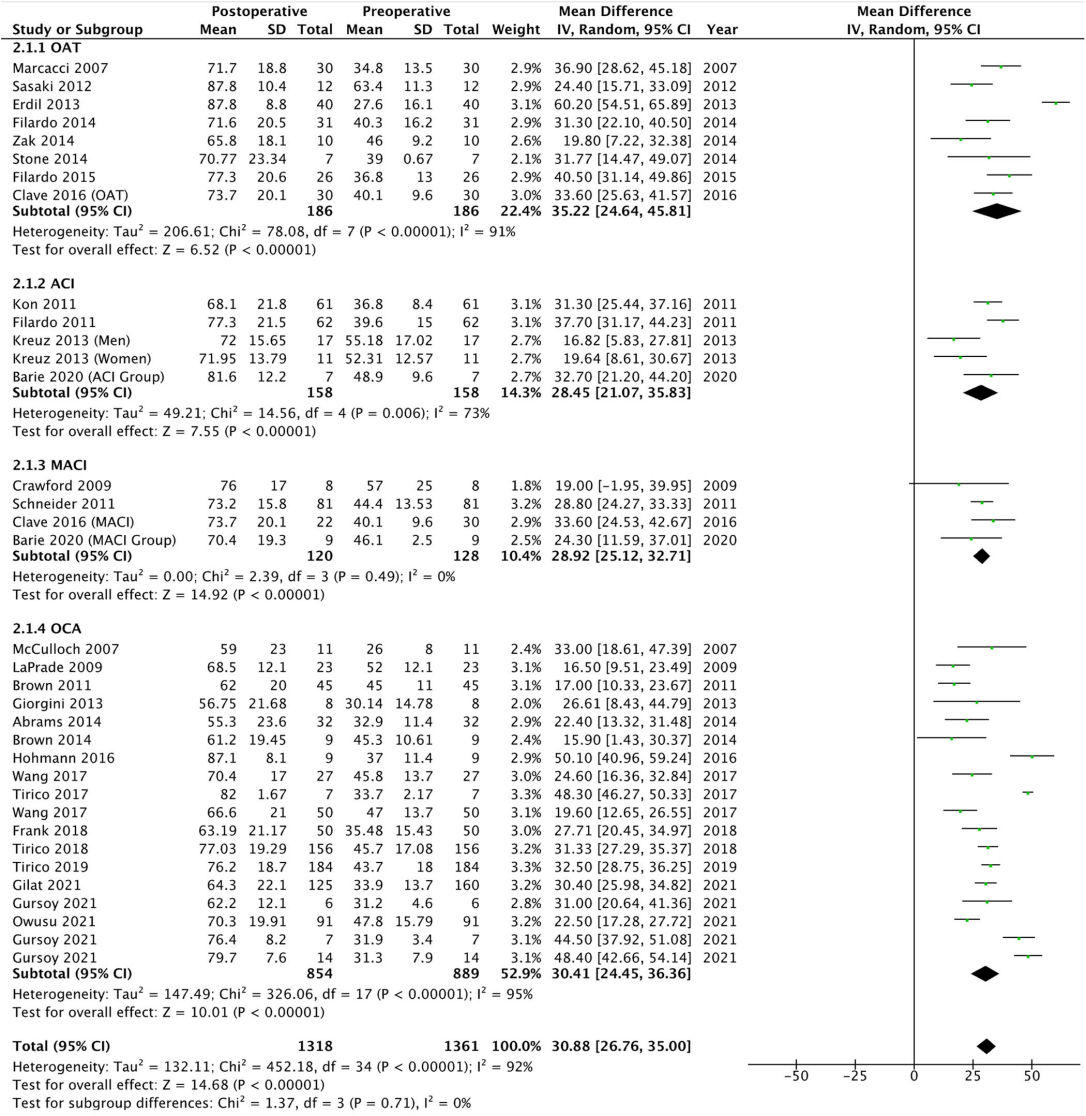
Forest plot showing mean improvement in International Knee Documentation Committee scores from preoperative to postoperative in patients treated for cartilage defects at the level of the tibiofemoral joint.

The subjective IKDC score was reported in 29 studies (61.7%), representing 1318 patients, with a mean score at final follow‐up of 71.9 ± 20.2 (range: 55.3–87.8). Patients treated using ACI reported a mean score of 73.0 ± 20.6 (range: 68.1–81.6), those treated using MACI reported a mean score of 74.6 ± 17.5 (range: 70.4–78.2), those treated using OAT reported a mean score of 76.5 ± 19.0 (range: 60.0–87.8) and those treated using OCA reported a mean score of 70.3 ± 20.6 (range: 55.3–87.1). Patients undergoing ACI reported a 28.5 point improvement [95% CI, 21.1, 35.8] (*p* < 0.05) from preoperative to postoperative IKDC scores, those undergoing MACI reported a 28.9 point improvement [95% CI, 25.1, 32.7] (*p* < 0.05), those undergoing OAT showed a 35.2 point improvement [95% CI, 24.6, 45.8] (*p* < 0.05), while those undergoing OCA reported a 30.4 point improvement [95% CI, 24.5, 36.4] (*p* < 0.05) (*p*‐subgroup difference > 0.05) (Figure 3).

### Tegner score

**Figure 4:**
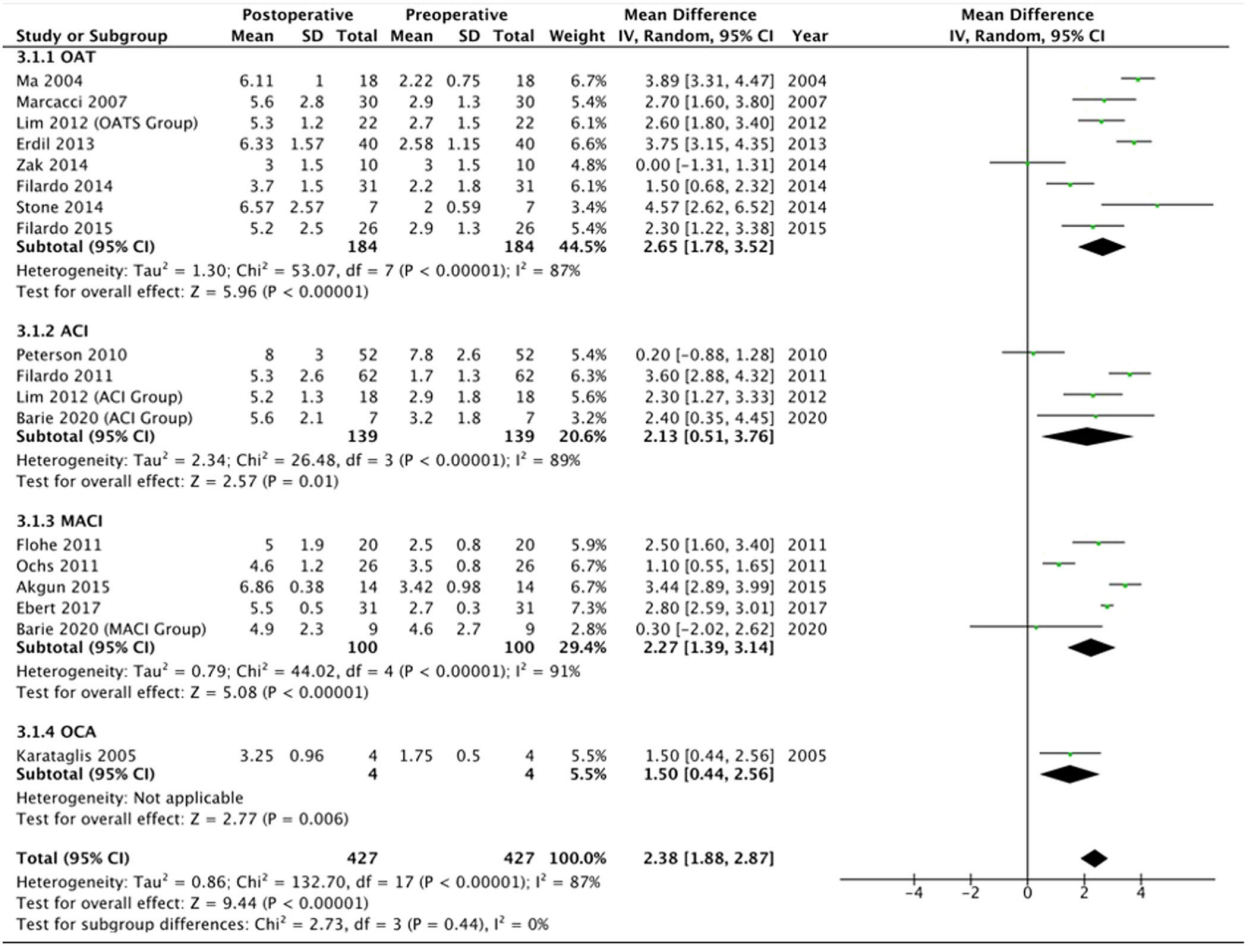
Forest plot showing mean improvement in Tegner scores from preoperative to postoperative in patients treated for cartilage defects at the level of the tibiofemoral joint.

The Tegner score was reported in 16 studies (34.0%), representing 494 patients, with a mean score at final follow‐up of 5.8 ± 2.3 (range: 3.0–8.0). Patients treated using ACI reported a mean score of 6.3 ± 2.9 (range: 5.3–8.0), those treated using MACI reported a mean score of 5.3 ± 1.4 (range: 4.6–6.9), those treated using OAT reported a mean score of 5.7 ± 2.0 (range: 3.0–7.0) and one study reported on patients undergoing OCA with a mean score of 3.3 ± 1.0. Patients undergoing ACI showed a 2.1 point improvement [95% CI, 0.5, 3.8] (*p* < 0.05) from preoperative to last follow‐up Tegner scores, those undergoing MACI reported a 2.3 point improvement [95% CI, 1.4, 3.1] (*p* < 0.05), those undergoing OAT reported a 2.7 point improvement [95% CI, 1.8, 3.5] (*p* < 0.05), while those undergoing OCA showed a 1.5 point improvement [95% CI, 0.4, 2.6] (*p* < 0.05) (*p*‐subgroup difference > 0.05) (Figure 4).

### General VAS

**Figure 5:**
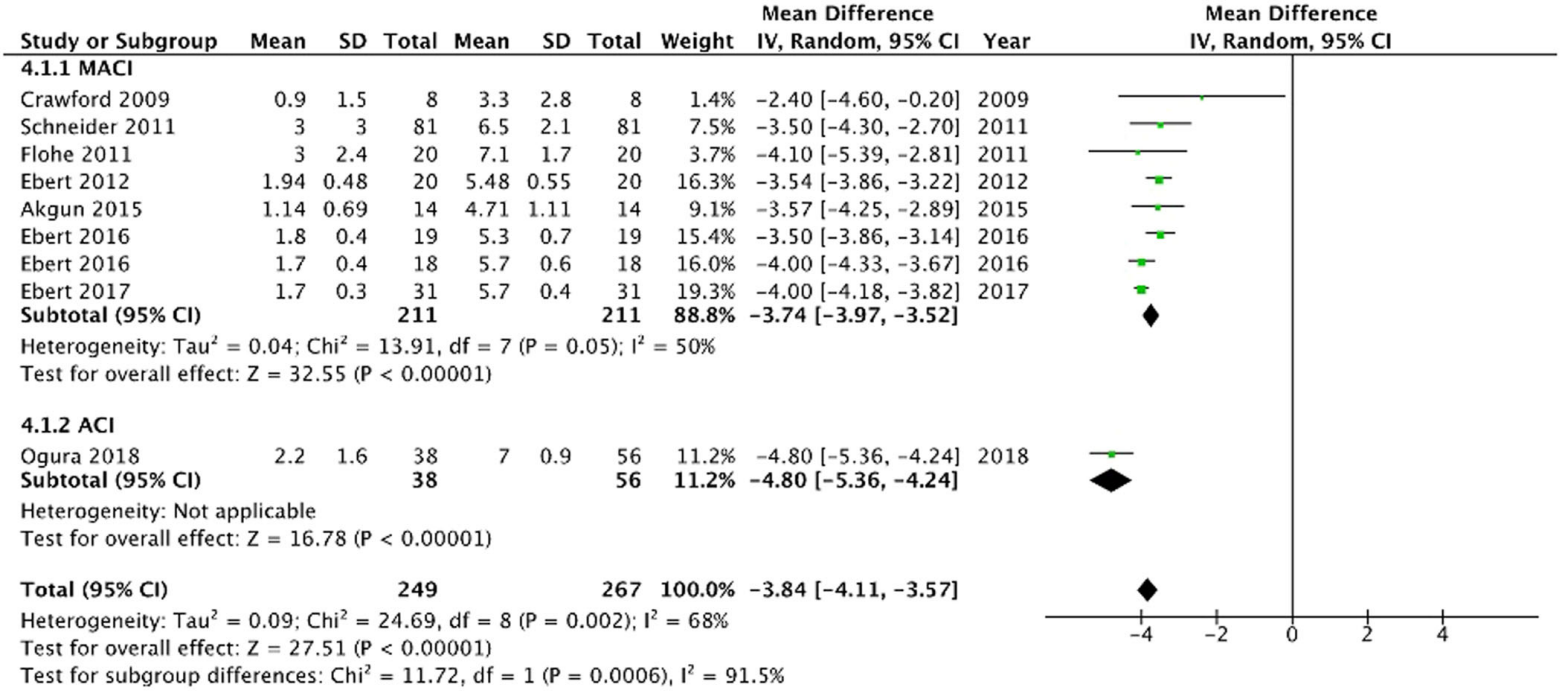
Forest plot showing mean improvement in Visual Analogue Scale scores from preoperative to postoperative in patients treated for cartilage defects at the level of the tibiofemoral joint.

The general VAS score was reported in eight studies (17.0%), representing 249 patients, with a mean score at final follow‐up of 2.3 ± 2.1 (range: 0.9–3.2). Only one study reported on General VAS for patients treated using ACI with a mean score of 2.2 ± 1.6 while those treated with MACI reported a mean score of 2.3 ± 2.1 (range: 0.9–3.2). Studies assessing OAT or OCA did not include VAS as an outcome. Patients undergoing ACI showed a 4.8 point improvement [95% CI, 4.24, 5.36] (*p* < 0.05) from preoperative to last follow‐up VAS scores, while those undergoing MACI showed a 3.7 point improvement [95% CI, 3.5, 4.0] (*p* < 0.05) (p‐subgroup difference > 0.05) (Figure 5).

The mean postoperative IKDC score for the OAT technique was 76.5 ± 19.0 (range: 60.0–87.8), surpassing the threshold PASS score of 75.9 [1]. In contrast, the ACI, MACI and OCA techniques did not meet this threshold. However, all four techniques exceeded the PASS thresholds of 70.0 for the Lysholm score and 3.5 for the Tegner score [3, 4].

### RoB assessment

For the nonrandomized studies (*n* = 41), the mean MINORS score was 12.8 (range: 8–21) (Table 4). Specifically, for the non‐comparative studies (*n *= 38), the mean score was 12.2 (range: 8–14), while the comparative studies (*n* = 3) had a mean score of 20.3 (range: 19–21). Furthermore, none of the included studies performed a prospective evaluation of sample size, with 29 studies (70.7%) not including consecutive patients, 11 studies (26.8%) not prospectively collecting data and 13 studies (31.7%) reporting a loss to follow‐up greater than 5%. The remaining criteria were adequately reported by all studies. For the randomized studies (*n* = 6), the mean JADAD score was 4.3 (range: 3.0–5.0) (Table 5).

**Table 4:** MINORS scores of the included nonrandomized studies.

Author year	MINORS items												
	1	2	3	4	5	6	7	8	9	10	11	12	Total
**Chow 2004** [13]	**2**	**2**	**2**	**2**	**2**	**2**	**1**	**0**	**‐**	**‐**	**‐**	**‐**	**13**
**Ma 2004** [42]	**2**	**0**	**0**	**2**	**2**	**2**	**2**	**0**	**‐**	**‐**	**‐**	**‐**	**10**
**Ozturk 2006** [49]	**2**	**0**	**0**	**2**	**2**	**2**	**0**	**0**	**‐**	**‐**	**‐**	**‐**	**8**
**Marcacci 2007** [44]	**2**	**0**	**2**	**2**	**2**	**2**	**2**	**0**	**‐**	**‐**	**‐**	**‐**	**12**
**Crawford 2009** [15]	**2**	**0**	**2**	**2**	**2**	**2**	**1**	**0**	**‐**	**‐**	**‐**	**‐**	**11**
**Peterson 2010** [50]	**2**	**0**	**1**	**2**	**2**	**2**	**1**	**0**	**‐**	**‐**	**‐**	**‐**	**10**
**Schneider 2011** [52]	**2**	**2**	**2**	**2**	**2**	**2**	**1**	**0**	**‐**	**‐**	**‐**	**‐**	**13**
**Kon 2011** [36]	**2**	**2**	**2**	**2**	**2**	**2**	**1**	**0**	**‐**	**‐**	**‐**	**‐**	**13**
**Filardo 2011** [22]	**2**	**2**	**2**	**2**	**2**	**2**	**1**	**0**	**‐**	**‐**	**‐**	**‐**	**13**
**Flohe 2011** [25]	**2**	**2**	**2**	**2**	**2**	**2**	**2**	**0**	**2**	**2**	**1**	**2**	**21**
**Sasaki 2012** [51]	**2**	**0**	**0**	**2**	**2**	**2**	**0**	**0**	**‐**	**‐**	**‐**	**‐**	**8**
**Ebert 2012** [17]	**2**	**2**	**2**	**2**	**2**	**2**	**2**	**0**	**‐**	**‐**	**‐**	**‐**	**14**
**Emre 2013** [20]	**2**	**0**	**1**	**2**	**2**	**2**	**0**	**0**	**‐**	**‐**	**‐**	**‐**	**9**
**Erdil 2013** [21]	**2**	**2**	**0**	**2**	**2**	**2**	**1**	**0**	**‐**	**‐**	**‐**	**‐**	**11**
**Kreuz 2013** [37]	**2**	**0**	**2**	**2**	**2**	**2**	**2**	**0**	**2**	**2**	**1**	**2**	**19**
**Basad 2014** [7]	**2**	**2**	**2**	**2**	**2**	**2**	**2**	**0**	**‐**	**‐**	**‐**	**‐**	**14**
**Filardo 2014** [23]	**2**	**0**	**2**	**2**	**2**	**2**	**2**	**0**	**‐**	**‐**	**‐**	**‐**	**12**
**Zak 2014** [61]	**2**	**1**	**1**	**2**	**2**	**2**	**1**	**0**	**‐**	**‐**	**‐**	**‐**	**11**
**Filardo 2015** [24]	**2**	**2**	**2**	**2**	**2**	**2**	**1**	**0**	**‐**	**‐**	**‐**	**‐**	**13**
**Bhattacharjee 2016** [8]	**2**	**0**	**2**	**2**	**2**	**2**	**2**	**0**	**‐**	**‐**	**‐**	**‐**	**12**
**Ebert 2017** [18]	**2**	**0**	**2**	**2**	**2**	**2**	**1**	**0**	**‐**	**‐**	**‐**	**‐**	**11**
**Ogura 2018** [47]	**2**	**0**	**1**	**2**	**2**	**2**	**2**	**0**	**‐**	**‐**	**‐**	**‐**	**11**
**Abrams 2014** [1^1^]	**2**	**1**	**1**	**2**	**2**	**2**	**2**	**0**	**‐**	**‐**	**‐**	**‐**	**12**
**Brown 2011** [10]	**2**	**1**	**2**	**2**	**2**	**2**	**2**	**0**	**‐**	**‐**	**‐**	**‐**	**13**
**Brown 2014** [11]	**2**	**1**	**2**	**2**	**2**	**2**	**2**	**0**	**‐**	**‐**	**‐**	**‐**	**13**
**Frank 2018** [26]	**2**	**1**	**2**	**2**	**2**	**2**	**2**	**0**	**2**	**2**	**2**	**2**	**21**
**Gilat 2021** [27]	**2**	**2**	**2**	**2**	**2**	**2**	**2**	**0**	**‐**	**‐**	**‐**	**‐**	**14**
**Giorgini 2013** [29]	**2**	**1**	**1**	**2**	**2**	**2**	**2**	**0**	**‐**	**‐**	**‐**	**‐**	**12**
**Gursoy 2021** [31]	**2**	**1**	**1**	**2**	**2**	**2**	**2**	**0**	**‐**	**‐**	**‐**	**‐**	**12**
**Hohmann 2016** [33]	**2**	**1**	**2**	**2**	**2**	**2**	**2**	**0**	**‐**	**‐**	**‐**	**‐**	**13**
**Karataglis 2005** [35]	**2**	**1**	**2**	**2**	**2**	**2**	**2**	**0**	**‐**	**‐**	**‐**	**‐**	**13**
**LaPrade 2009** [39]	**2**	**2**	**2**	**2**	**2**	**2**	**2**	**0**	**‐**	**‐**	**‐**	**‐**	**14**
**McCulloch 2007** [45]	**2**	**2**	**2**	**2**	**2**	**2**	**2**	**0**	**‐**	**‐**	**‐**	**‐**	**14**
**Owusu 2021** [48]	**2**	**1**	**2**	**2**	**2**	**2**	**2**	**0**	**‐**	**‐**	**‐**	**‐**	**13**
**Scorrano 2004** [53]	**2**	**1**	**2**	**2**	**2**	**2**	**2**	**0**	**‐**	**‐**	**‐**	**‐**	**13**
**Stone 2014** [54]	**2**	**1**	**2**	**2**	**2**	**2**	**2**	**0**	**‐**	**‐**	**‐**	**‐**	**13**
**Takazawa 2012** [55]	**2**	**1**	**2**	**2**	**2**	**2**	**2**	**0**	**‐**	**‐**	**‐**	**‐**	**13**
**Tirico 2017** [56]	**2**	**1**	**2**	**2**	**2**	**2**	**2**	**0**	**‐**	**‐**	**‐**	**‐**	**13**
**Tirico 2018** [57]	**2**	**1**	**2**	**2**	**2**	**2**	**2**	**0**	**‐**	**‐**	**‐**	**‐**	**13**
**Tirico 2019** [58]	**2**	**1**	**2**	**2**	**2**	**2**	**2**	**0**	**‐**	**‐**	**‐**	**‐**	**13**
**Wang 2017** [60]	**2**	**1**	**2**	**2**	**2**	**2**	**2**	**0**	**‐**	**‐**	**‐**	**‐**	**13**
**2**	**Methodological requirement reported and adequate**												
**1**	**Methodological requirement reported but inadequate**												
**0**	**Methodological requirement not reported**												

*Note*: The MINORS score consists of 12 questions, with each question scored as a 0 if not reported (red), 1 if reported but inadequate (yellow) or 2 if reported and adequate (green). The maximum score is 16 for noncomparative studies and 24 for comparative studies. MINORS items: 1 = a clearly stated aim; 2 = inclusion of consecutive patients; 3 = prospective collection of data; 4 = endpoints appropriate to the aim of the study; 5 = unbiased assessment of the study endpoint; 6 = follow‐up period appropriate to the aim of the study; 7 = loss to follow‐up <5%; 8 = prospective evaluation of the study size; 9 = a control group having the gold standard intervention; 10 = contemporary groups; 11 = baseline equivalence of groups; 12 = statistical analysis adapted to the study design.

Abbreviation: MINORS, methodological index for nonrandomized studies.

**Table 5:** Jadad scores of the included randomized controlled trials.

JADAD score				
Author year	Randomization	Blinding	Account of all patients	Total
**Akgun 2015** [3]	2	0	1	3
**Barie 2020** [6]	2	0	1	3
**Lim 2012** [40]	2	2	1	5
**Clave 2016** [14]	2	2	1	5
**Ebert 2017** [16]	2	2	1	5
**Gudas 2012** [30]	2	2	1	5

## DISCUSSION

The most important finding from this study was that all four surgical techniques reported significant improvements of the Lysholm, subjective IKDC and Tegner at final follow‐up when compared to preoperative values. There was no significant difference in improvements between procedures.

The MCID refers to the smallest change in a score that a patient perceives as beneficial enough to warrant changing their management [34]. Importantly, the MCID represents the smallest change in PROMs that a patient would recognize as an improvement or a decline from their initial state [34, 38]. The improvements observed in all four treatments analyzed in this study were greater than the MCID thresholds for Lysholm score (8.9), subjective IKDC (16.7), Tegner score (1.0) and VAS (1.37) [1, 2].

Although MCID is frequently used as a metric to assess clinically significant differences, it is limited due to its focus on amount of change, rather than the patient's final symptom state. Therefore, a patient with a poor preoperative baseline could surpass the MCID, while still maintaining poor postoperative outcomes. As a result, the use of the patient's PASS has gained popularity. In contrast to MCID, PASS represents an absolute postoperative score, indicating the point at which a patient is expected to reach a satisfactory level of symptoms. This makes PASS a more accurate indicator of clinical outcomes, as it does not depend on the preoperative baseline but instead evaluates the patient's overall satisfaction [43].

The mean postoperative IKDC score for the OAT technique was 76.5 ± 19.0 (range: 60.0–87.8), surpassing the PASS score of 75.9 [1]. The ACI, MACI and OCA techniques did not meet this threshold. All four techniques surpassed the PASS threshold of 70 for the Lysholm score and the PASS threshold of 3.5 for the Tegner score [3, 4]. This suggests that all four of these surgical techniques can address cartilage defects of the tibiofemoral joint in a manner that provides substantial symptomatic relief.

Because all four surgical techniques produce favourable clinical outcomes, specific patient and cartilage defects characteristics must be considered when selecting a treatment option. For instance, ACI and MACI can be used to treat cartilage defects up to 10 cm^2^, whereas OAT is more suitable for smaller defects between 1 and 4 cm^2^ [5]. ACI and MACI are often indicated in patients with full‐thickness cartilage defects surrounded by healthy hyaline cartilage who have previously failed debridement or other conservative measures. Despite MACI and ACI having clinically similar results, MACI may be preferred by patients due to the smaller incision [5]. OAT is indicated for cartilage defects involving subchondral bone, as donor plugs consist of both cartilage and bone tissue. While multiple donor plugs may be used for larger cartilage defects, this carries increased risk of donor site morbidity [21]. Conversely, OCA uses donor allograft cartilage, eliminating the risk of donor site morbidity, which makes it preferable to treat cartilage defects larger than 3 cm^2^. Both OAT and OCA are suitable for young, active patients, while OCA is also effective for older, more sedentary patients [12].

Ginesin et al. conducted a systematic review assessing patellofemoral cartilage defects and found that OAT, OCA, ACI and MACI all yielded significant improvements in PROMs with low complication rates [28]. While their study focused solely on patellofemoral defects, our study examined tibiofemoral joint cartilage defects. They found OAT to be effective for smaller cartilage defects, particularly those <2 cm². Similarly, we found OAT to be effective for small tibiofemoral cartilage defects, with a mean size of 2.9 ± 1.4 cm², further reinforcing its suitability for smaller defects. Ginesin et al. also emphasized that both ACI and MACI were effective for larger patellofemoral defects, and our study supports this by demonstrating that these techniques also deliver excellent results for tibiofemoral cartilage defects [28]. Overall, their review highlights the effectiveness of these surgical techniques for patellofemoral defects across various lesion sizes, with our findings extending these conclusions to the tibiofemoral joint.

Our study has several limitations that mainly stem from the nature of the included studies. While we included studies with levels of evidence ranging from 1 to 4, the majority were of level 4, with only three studies reported as level 1 evidence. Moreover, we included 16 studies defined as ‘low’ quality following the MINORS criteria, indicating a high RoB which could compromise the overall validity of our conclusions. It is also important to note the heterogeneity across the analyzed groups, including differences in patient demographics, defect size, defect location and indications for each procedure, rendering it more difficult to directly compare treatment procedures. Furthermore, many comparisons across studies were conducted indirectly from case series data, rather than from direct randomized controlled trials, which introduces additional biases. Indirect comparisons are less reliable and may affect the validity of our findings, particularly, when comparing the clinical outcomes of different surgical techniques. While the included studies did show significant pre–post improvements in outcomes, future studies should aim to use randomized controlled trials to provide more robust, direct comparisons and reduce the influence of these biases. In addition, we recognize that the indications for each procedure may differ depending on the defect size and location and that direct comparisons between procedures need to take this into consideration.

## CONCLUSIONS

In conclusion, this systematic review and meta‐analysis indicate that ACI, MACI, OAT and OCA all result in significant improvements in knee function and pain for cartilage defects of the tibiofemoral joint. The OAT technique surpassed the PASS threshold for the IKDC score and all four techniques surpassed the PASS threshold for Tegner and Lysholm scores. Additionally, each procedure met the MCID for each clinical outcome. Therefore, when selecting a procedure, patient and cartilage defect characteristics should be assessed to determine the best technique for each individual patient.

## AUTHOR CONTRIBUTIONS

*Conceptualization*: Raffaella Russo and Filippo Familiari. *Literature search and data analysis*: Joseph E. Nassar, Grace Guerin and Taidhgin Keel. *Drafted and/or critically revised the work*: Joseph E. Nassar, Grace Guerin, Taidhgin Keel, Raffaella Russo, Filippo Familiari, Luke V. Tollefson and Robert F. LaPrade.

## CONFLICTS OF INTEREST STATEMENT

R. F. L. has received consulting fees from Ossur, Smith & Nephew and Responsive Arthroscopy; royalties from Ossur, Smith & Nephew, Elsevier and Arthrex; research support from Ossur and Smith & Nephew and support for education from Foundation Medicine. The remaining authors declare no conflict of interest.

## ETHICS STATEMENT

The authors have nothing to report.

## Data Availability

The raw data and materials supporting the findings of this study are available upon request from the corresponding author.
